# Megapinosomes and homologous structures in hematopoietic cells

**DOI:** 10.1007/s00418-022-02124-x

**Published:** 2022-07-12

**Authors:** Andrea Bauer, Giada Frascaroli, Paul Walther

**Affiliations:** 1 Central Facility for Electron Microscopy, Ulm University, Albert-Einstein-Allee 11, 89081 Ulm, Germany; 2 HPI, Leibniz Institute for Experimental Virology, 20251 Hamburg, Germany

**Keywords:** M macrophages, Megapinosomes, Megakaryocytes, Blood platelets, High-pressure freezing, Electron microscopy, STEM tomography

## Abstract

Megapinosomes are endocytic organelles found in human macrophage colony-stimulating factor (M-CSF) monocyte-derived M macrophages. They are large (several microns) and have a complex internal structure that is connected with the cytosol and consists of interconnected knots and concave bridges with sizes in the range of 100 nm. We called this structure trabecular meshwork. The luminal part of the megapinosome can be connected with luminal tubules and cisterns that form the megapinosome complex. The structures are especially well visible in scanning electron tomography when macrophages are prepared by high-pressure freezing and freeze substitution. Our research received a new impulse after studying the literature on hematopoietic cells, where very similar, most likely homologous, structures have been published in peritoneal macrophages as well as in megakaryocytes and blood platelets. In platelets, they serve as membrane storage that is used for structural changes of platelets during activation.

**Supplementary Information:**

The online version contains supplementary material available at 10.1007/s00418-022-02124-x.

## Introduction

The megapinosome is a large endocytic structure that we observed in human M macrophages (Bauer et al. 2016, 2020). Under electron microscopy, the megapinosome can be clearly distinguished from other endocytic structures and other cell organelles with the help of morphological criteria. The cell-biological functions of the megapinosome remain largely enigmatic. Probably, megapinosomes ensure storage of surface membranes that can be promptly made available when a macrophage needs to change shape to move through a tissue, to uptake extracellular material or dead cells, as well as to fight against microbes. Structures similar to megapinosomes can also be found in other hematopoietic cells, such as peritoneal macrophages (labyrinth), megakaryocytes (demarcation membrane system), and blood platelets (open canalicular system). We assume that these structures are homologous to megapinosomes, although their functions may be different. In the following, we present an exact structural description of the megapinosome and compare it with similar structures of hematopoietic cells as they appear in literature.

## Megapinosomes in human M macrophages

**Fig. 1:**
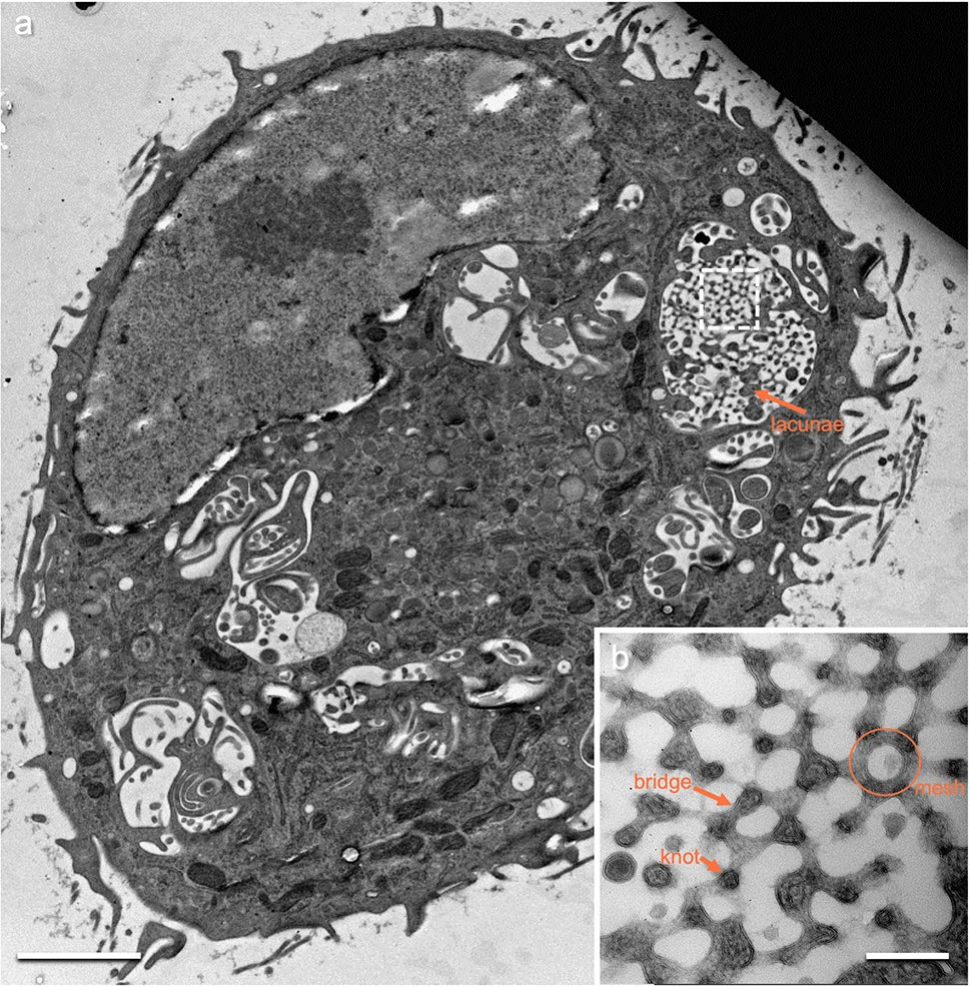
M macrophage high-pressure frozen, freeze-substituted, and imaged in TEM, showing a megapinosome and elements of megapinosome complexes. **b** Higher magnification of the megapinosome in **a**, showing the dense trabecular meshwork topologically equivalent to the cytosol and the bright luminal part. The two parts are separated by a membrane. The trabecular meshwork consists of interconnected knots and concave bridges. In the 2D TEM image, one knot connects via bridges with three adjacent knots. Usually, five to six bridges form a mesh surrounding a lumen with an average diameter of 110 nm. Another cytosolic structure within the megapinosome are the lacunae. The scale bars represent 2 µm in **a** and 200 nm in **b**

Human M macrophages are hematopoietic cells with size from 20 to 60 µm and serve as key components of the innate immune system. One role beyond innate immunity is homeostasis, tissue repair and remodeling, and clearance of cellular debris (Zhang et al. 2021; Herzog et al. 2019). For this purpose, they absorb large amounts of fluid, clarify it, and release the processed fluid back into the extracellular space (Alberts et al. 2014). To identify endocytic structures, we incubated M macrophages (derived from peripheral blood monocytes) with 10-nm colloidal gold. Compartments that contained gold particles were considered to be endocytic. When analyzed under electron microscopy (after high-pressure freezing and freeze substitution), structures are visible that contain colloidal gold particles but appear very different from canonical endocytic structures such as endosomes or macropinosomes (Fig. 1a). We called these structures megapinosomes and megapinosome complex (Bauer et al. 2016, 2020). In contrast to macropinosomes and endosomes, megapinosomes have a very prominent electron-dense internal structure, that is, the trabecular meshwork (Fig. 1b), which is topologically equivalent to and connected with the cytosol. The luminal part filled with extracellular fluid is electron lucent and separated by a membrane from the trabecular meshwork. The trabecular meshwork consists of interconnected knots and concave bridges. The knots have a diameter between 60 and 90 nm. The bridges between two knots have a length of up to 130 nm. Most concave bridges have a diameter of 30–40 nm at their thinnest point. In two-dimensional (2D) transmission electron microscopy (TEM) images, one knot connects via bridges with three adjacent knots. Usually, five to six bridges form a mesh surrounding a lumen with an average diameter of 110 nm. An additional cytosolic structure in the megapinosome are the lacunae (Bauer et al. 2020). Notably, the structural components of the trabecular meshwork (knots, bridges, and meshes) were similar in size in all megapinosomes examined, regardless of the overall size of the megapinosome. The trabecular meshwork serves as a marker for a megapinosome under electron microscopy.

**Fig. 2:**
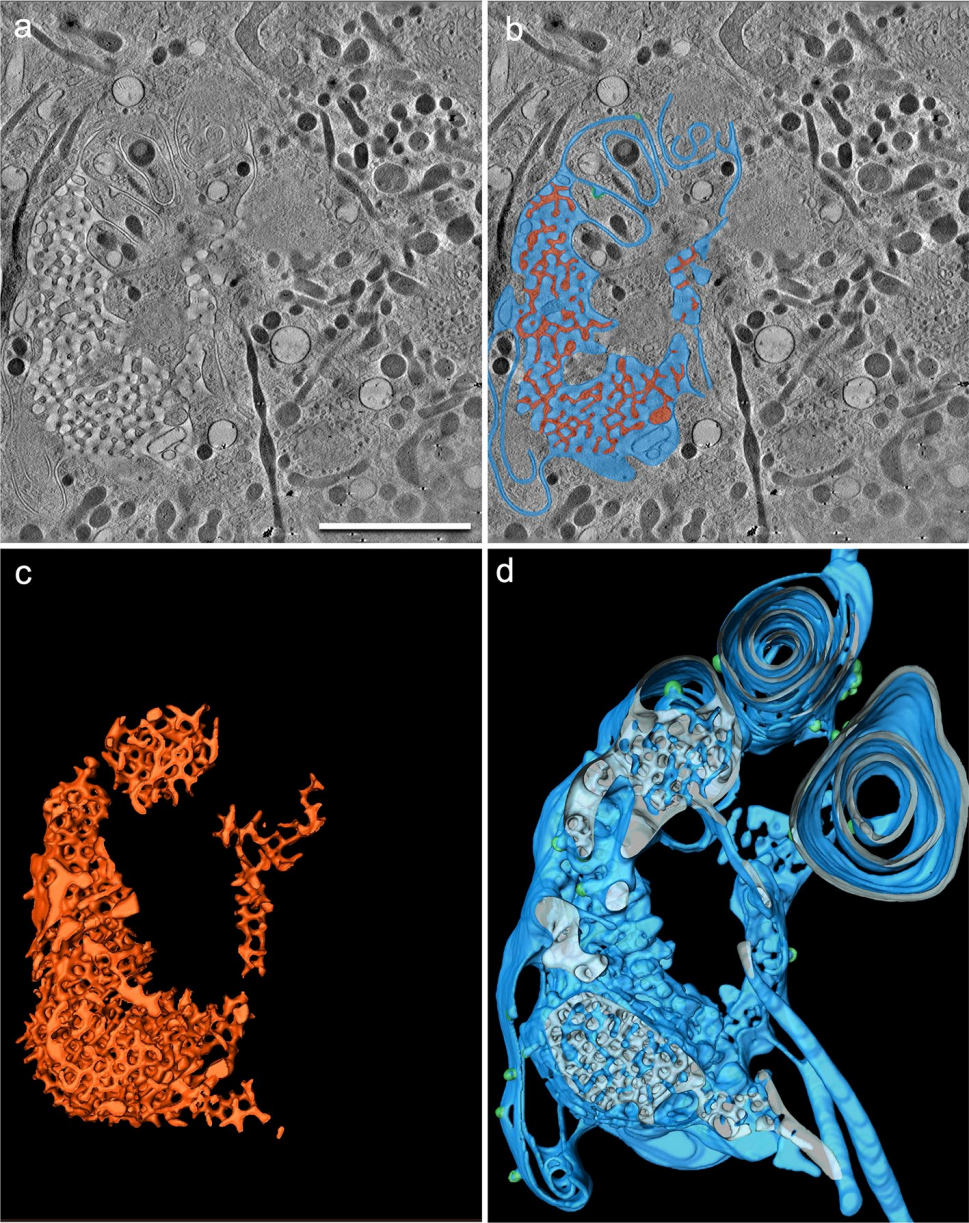
Megapinosome complex in 3D, obtained from STEM tomography data. **a** Virtual section showing the trabecular meshwork on top. **b** The same virtual section with the trabecular meshwork that is part of the cytosol in red, and the luminal part in blue. **c** The segmented (red) trabecular meshwork in 3D. **d** The corresponding highly branched channel system of the luminal part of the megapinosome in 3D. It is connected with cisterns with a continuous 30-nm gap, as well as with tubules with an average diameter of 165 nm. The trabecular meshwork as well as the luminal part are continuous, meaning that, from each point of the trabecular meshwork (in red), each other point in the trabecular meshwork can be reached without passing through a membrane, and the same is true for the luminal part in blue. The scale bar represents 2 µm; all images at the same magnification. A movie of these data is available in the Supplementary Material

**Fig. 3:**
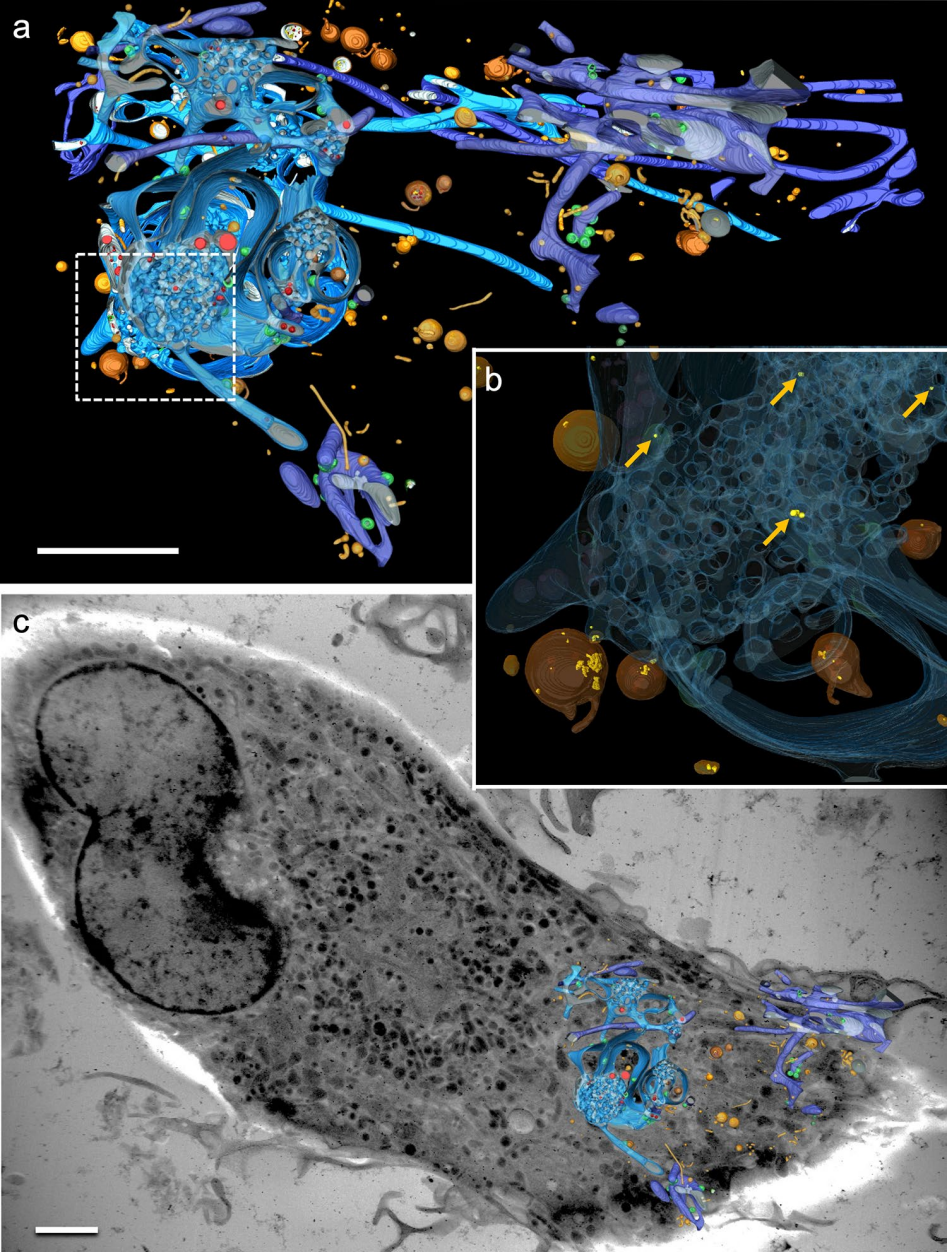
A large portion of a megapinosome complex segmented from a total of 17 **a** and 9 **c** STEM tomograms from two adjoint 600-nm-thick sections. The bright-blue parts form a continuum. The dark-blue parts are not connected with the bright-blue parts in the segmented area. They may, however, be connected in another section plane. In brown, the structures are segmented that are endocytic but not connected to the megapinosome. Many of these structures most likely represent canonical endosomes. **b** is an enlarged portion of the encircled area in **a**. The blue luminal part as well as the brown endosomes are transparent so that the yellow endocytosed gold particles become visible. The gold particles are markers for endocytic structures. The yellow arrows depict the gold particles in the luminal part of the megapinosome. The scale bars represent 2 µm

Three-dimensional (3D) data obtained from scanning transmission electron (STEM) tomography from a megapinosome complex are presented in Fig. 2. The segmentations of the trabecular meshwork (**c**, red) and of the luminal part of the megapinosome (**d**, blue) show many similarities. Both structures are highly structured but continuous, meaning that, from each point of the trabecular meshwork (in red), each other point in the trabecular meshwork can be reached without passing through a membrane, and the same is true for the highly branched channel system of the luminal part in blue. (This is not a trivial finding and is usually not the case in corresponding cellular structures; e.g., vesicles are in most cases isolated spheres in the cytosol, so the 3D structures of the vesicles and of the surrounding cytosol look quite different.) Clathrin-coated pits (Fig. 2d, green) often bud at the outer membrane area of the megapinosome or on the lacunae, but not in the trabecular meshwork. The trabecular meshwork spans the entire megapinosome with its irregular shape and is connected to the cytosol at several points. The luminal portion of the megapinosome (Fig. 2b, d, blue) can expand into membrane extensions. We classified all luminal structures that are connected with the megapinosome’s lumen as megapinosome complex. This large complex consists of tubules and cisterns as explained below and depicted in Fig. 2d. The continuous membranes of the cisterns form oval, bowl-like curved structures with a constant-sized luminal gap of 30 nm. The cisterns with such a constant gap often extend over the entire height of the tomogram of about 600 nm. This is especially well visible in the movie in the Supplementary Material. In the serial tomograms shown in Fig. 3, the cisterns could even be tracked over both serial sections (1200 nm). This constant gap is rather unique among cell organelles; Golgi apparatus or endoplasmic reticulum never show such a constant cisternal gap. Several cisterns can arise from one megapinosome. They often form loops that originate at the outer part of the megapinosome and end on another part. The cisterns extend over large areas of the cell. The tubules of the megapinosome complex have an average diameter of 165 nm and can extend into the periphery of the macrophage; they can branch and reach several microns in length (Fig. 3, blue). This contrasts with canonical endosomes, which form small, self-contained spherical or thin tubular structures (Fig. 3, brown). The megapinosome complexes form large luminal structures that encompass large areas of a macrophage. In Fig. 3, all structures in bright blue are connected with each other. In Fig. 3b, the blue luminal part as well as the brown endosomes are transparent so that the yellow endocytosed gold particles become visible. Since we added the 10-nm gold to the medium 5 min before electron microscopic sample preparation (high-pressure freezing), the gold particles are markers for endocytic uptake. This strongly indicates that megapinosomes are endocytic structures, similar to the brown endosomes.

**Fig. 4:**
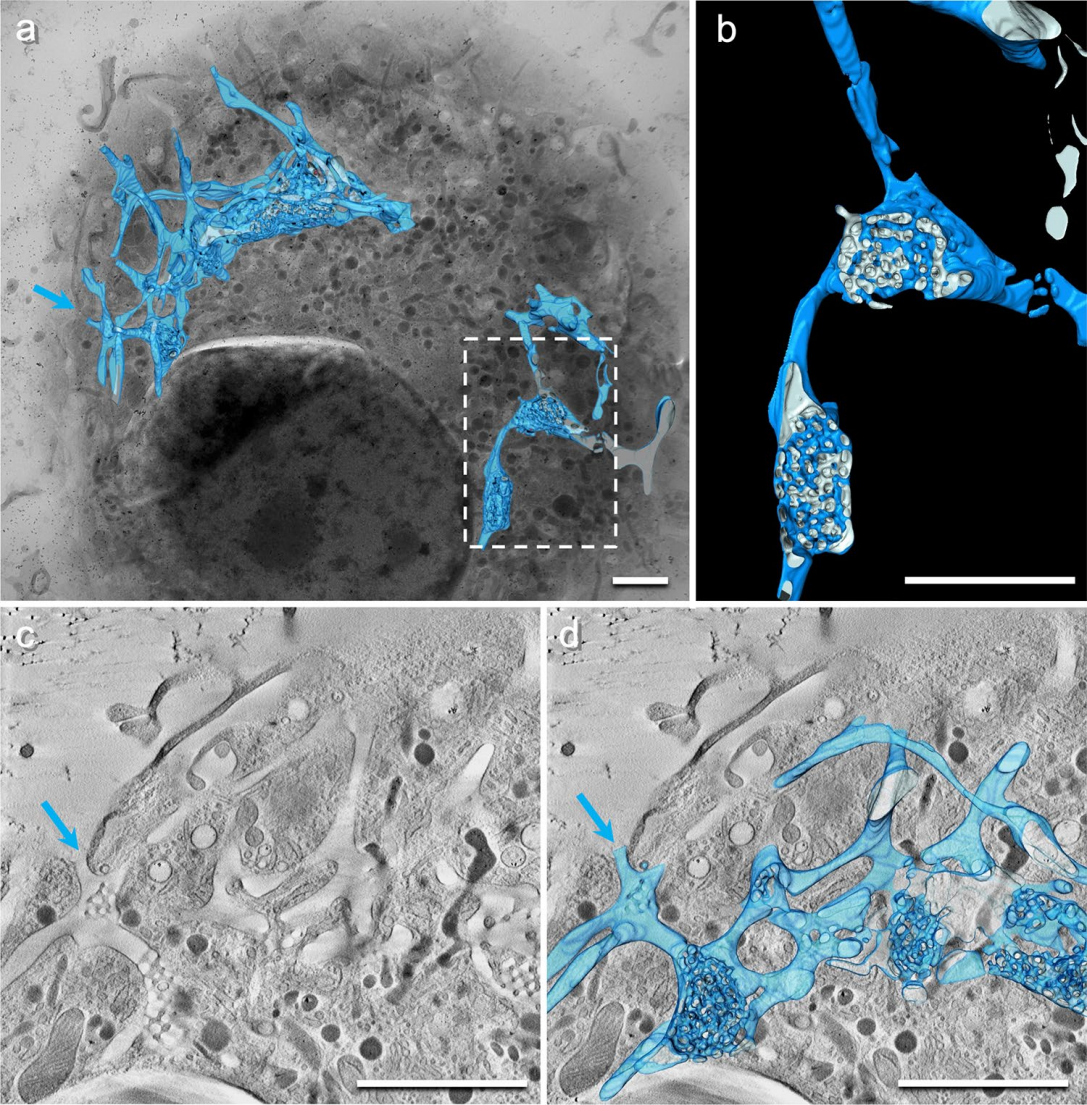
Several megapinosomes connected by tubules. Some of these tubules reach into the extracellular space. **a** is an overview of a M macrophage with the segmentation of a portion of the megapinosome complex. **b** Higher magnification of the encircled area in **a**, showing the connection of two megapinosomes with a tubule. **c** Virtual section of a tomogram where a tubule reaches the extracellular space (blue arrow). **d** Virtual section with the segmented luminal part of the megapinosome complex. The scale bars represent 2 µm

Tubules can connect different megapinosomes or lead into the extracellular space (Fig. 4, blue arrow). An M macrophage can contain several megapinosomes. We counted up to four megapinosomes or megapinsome complexes in one macrophage. Clathrin-coated pits bud not only on the megapinosome as mentioned above, but also frequently on the cisterns or on the tubules of the megapinosome complex structures.

**Fig. 5:**
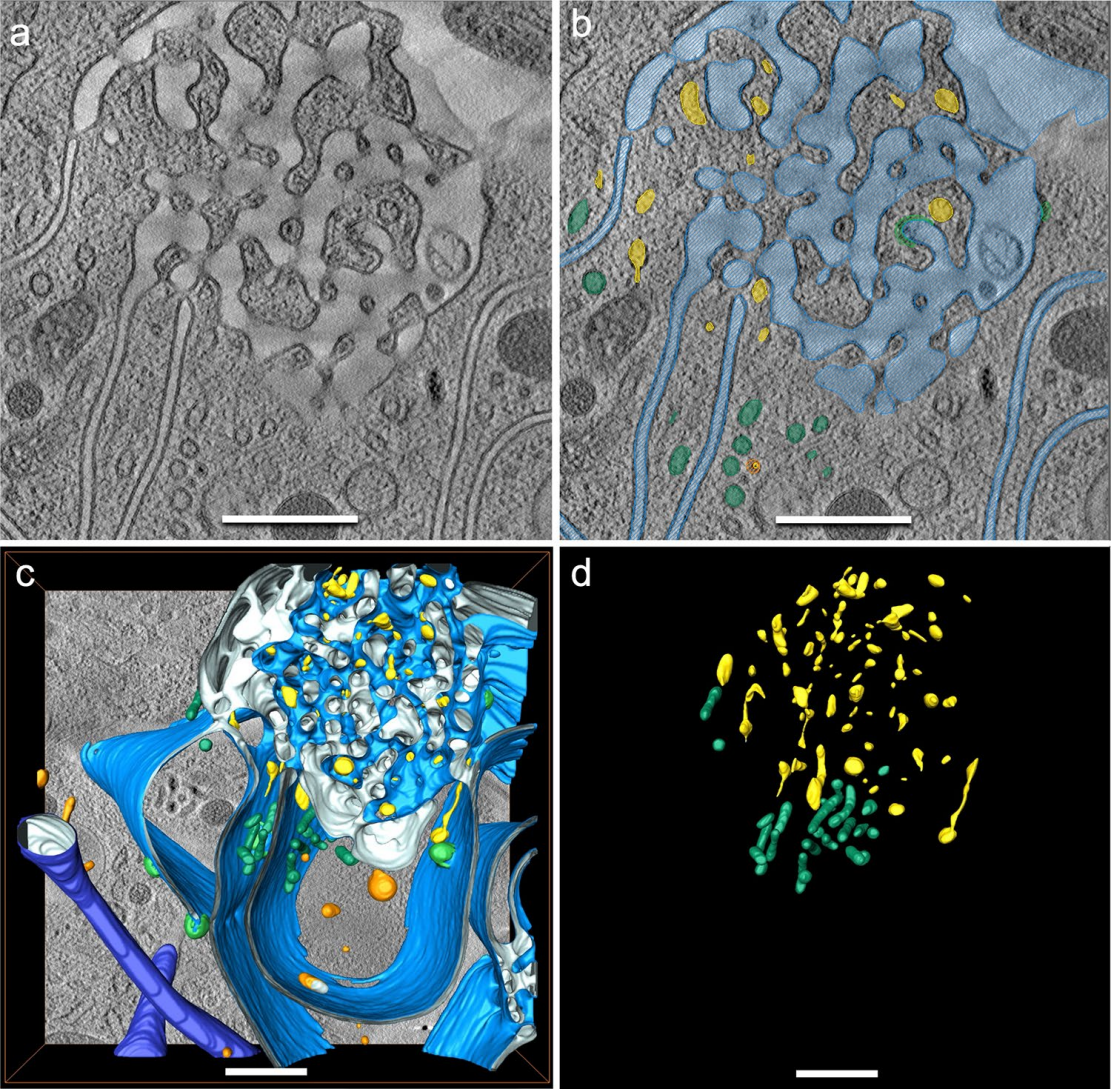
Portion of the megapinosome complex in Fig. 3. Beside the luminal part of the megapinosome (in blue), we found additional luminal structures that are not connected to the megapinosome lumen. They did not contain gold particles and are darker than the luminal parts of the megapinosome segmented in blue. Since we found two categories of these structures, we segmented the structures in the trabecular meshwork in yellow and the part adjacent to the trabecular meshwork in green. Whereas the green structure looks as expected for small tubular and vesicular structures, the yellow part shows polymorph shapes. Whether these structures are homologous to the “dense tubular system” in blood platelets needs to be further investigated. The scale bars represent 500 nm

Within the trabecular meshwork of the megapinosomes, we could see another fine structure. It appears to be enveloped by a membrane, but the content is considerably more electron dense than the luminal part of the megapinosome (Fig. 5, segmented in yellow). Since these luminal structures did not contain gold particles, they are not, by our definition, part of the endocytic system. Topologically, they could be homologous to the luminal structures in the “cytoplasmic part of the labyrinth” in peritoneal macrophages (Brederoo and Daems 1972), possibly deriving from the endoplasmic reticulum, and the “dense tubular system” in blood platelets (Heijnen and Korporaal 2017) (see below). The shape is polymorphic and often difficult to resolve in tomograms. This structure is not present in all knots and bridges. Some of these structures are interconnected, but they do not form a continuous tubular system as in blood platelets, as can be seen in Fig. 5d. Their polymorphic appearance allows them to be distinguished from other vesicular systems in the cell. Vesicles of similar diameter and content have been segmented in the vicinity of the megapinosome and show a distinctly vesicular or tubular structure (Fig. 5b–d, green structures).

## Megapinosome-homologous structures in other hematopoietic cells

Due to a hint from a colleague, we indeed found publications describing structures in hematopoietic cells that might be homologous to megapinosomes.

### Peritoneal macrophages contain megapinosome-like structures

In 1972, Brederoo and Daems published electron microscopic data of a previously undescribed organelle in resident peritoneal macrophages of guinea pigs, which they termed “labyrinth” (Figs. 18–25 in Brederoo and Daems 1972). They described it as an invagination of the cell surface forming “a globular complex of interconnected, membrane limited, channels,” where “the membrane of the labyrinth is continuous with the plasma membrane.” The labyrinths are connected with the cell surface and with other labyrinths with membrane “channels.” In consecutive sections, they were able to find up to three labyrinths in one cell. Further, they present a 3D reconstruction obtained by serial sectioning showing a labyrinth with connections (channels) to the cell surface (Fig. 20 in Brederoo and Daems 1972). This description is very similar to the megapinosome in human M macrophages. In addition, they observed budding “bristle coated vesicles” at the outer membrane area of the labyrinth, obviously representing clathrin-coated pits according to modern nomenclature. This agrees with our own studies of M macrophages, were we found numerous clathrin-coated pits in the megapinosome complex (Bauer et al. 2020). In addition, Brederoo and Daems (1972) used peroxidase reaction to label endoplasmic reticulum (ER) and related structures. They found the dark reaction product not only in the ER, as expected, but also in areas of the cytosolic part of the labyrinth, which we called trabecular meshwork (Fig. 21 in Brederoo and Daems 1972). This is a hint that there are areas of a second luminal system in the labyrinth that is in contact with the ER and that would be homologous to the “dense tubular system” (DTS) in blood platelets that is explained below. This would also correspond to the second luminal system that we observe in human M macrophages (Fig. 5, yellow). However, in our samples of M macrophages, these yellow structures are not connected to each other, as can be seen in the 3D reconstruction (Fig. 5d, yellow structure). The cytoplasmic part of the “labyrinth” that corresponds to the trabecular meshwork remains morphologically undescribed.

### Megakaryocytes and blood platelets contain megapinosome-like structures

Megakaryocytes grow in the bone marrow without dividing, resulting in the production of giant cells (70–160 µm). Within these giant cells, organelles organize into discrete domains that eventually become individual blood platelets which are fragments from the megakaryocytes. Megakaryocytes as well as blood platelets also contain megapinosome-like structures.

Megakaryocytes from mouse spleen were first investigated under electron microscopy by Yamada (1957). He observed an extensive membrane system inside the megakaryocytes. It was assumed that these areas will be released into the blood as newly formed platelets from inside the megakaryocyte during thrombopoiesis. This membrane system was named the “demarcation membrane system” (DMS) since it demarcates the future platelets from each other. It forms by invagination of the outer cell membrane, as shown by staining techniques (Behnke 1968; Zucker-Franklin and Petursson 1984). This model was expanded in the following years in various ways. The demarcation membrane system was morphologically described as tubular invaginations whose cavities are continuous with the extracellular space. In 1977, Thiele et al. (1977) described the invaginations of the demarcation membrane system as a “spongy system of flattened cisternae and tubules.” Schulze et al. (2006) confirmed the observation of tubules and flattened cisterns with 3D studies. Van Noord et al. (1973) described in an electron microscopic work “labyrinth-like structures” in cells of the megakaryocytic type, similar to the observations of Brederoo and Daems (1972) in resident peritoneal macrophages.

Since blood platelets (2–3 µm) sprout from megakaryocytes, they contain membrane infoldings similar to the demarcation membrane system of megakaryocytes (Zucker-Franklin et al. 1985). These membrane systems described from Behnke (1967) are mostly called “open canalicular system” (OCS). The expansion of the membrane system is very variable and can expand to a very large system throughout the platelet. In some places, the channels communicate with the extracellular space (Behnke 1967). The function of the structure is an internal membrane reservoir for changes in shape and spread of the blood platelets during activation (Heijnen and Korporaal 2017). However, this membrane storage is not only used for morphological changes; it is assumed that the membrane is also provided for secretion and endocytosis (White 1979; Zucker-Franklin et al. 1985; Escolar et al. 1989; Thon and Italiano 2012; Selvadurai and Hamilton 2018). In numerous publications, these structures have been described in 2D (Behnke 1967; White 1972) as well as in 3D (van Nispen tot Pannerden et al. 2010; Heijnen and Korporaal 2017; Pokrovskaya et al. 2020). In addition, a second luminal structure was found in the platelets, being called “dense tubular system,” which originates from the endoplasmic reticulum of the megakaryocyte and has no connection with the cell surface (Behnke 1967; Heijnen and Korporaal 2017). It is most likely homologous to the structures in peritoneal macrophages and to the yellow structure in our tomograms. In some areas, the OCS and DTS are interwoven and form a “membrane complex” (White 1972) that looks like the megapinosome in human M macrophages. It is striking that, in the membrane complex, the OCS in White’s and others’ TEM images are arranged in such a way that the cytosol between the OCS channels is very similar to our trabecular meshwork. Moreover, the diameter of the OCS channels in the membrane complex, about 110 nm, is similar to our mesh diameter.

**Fig. 6:**
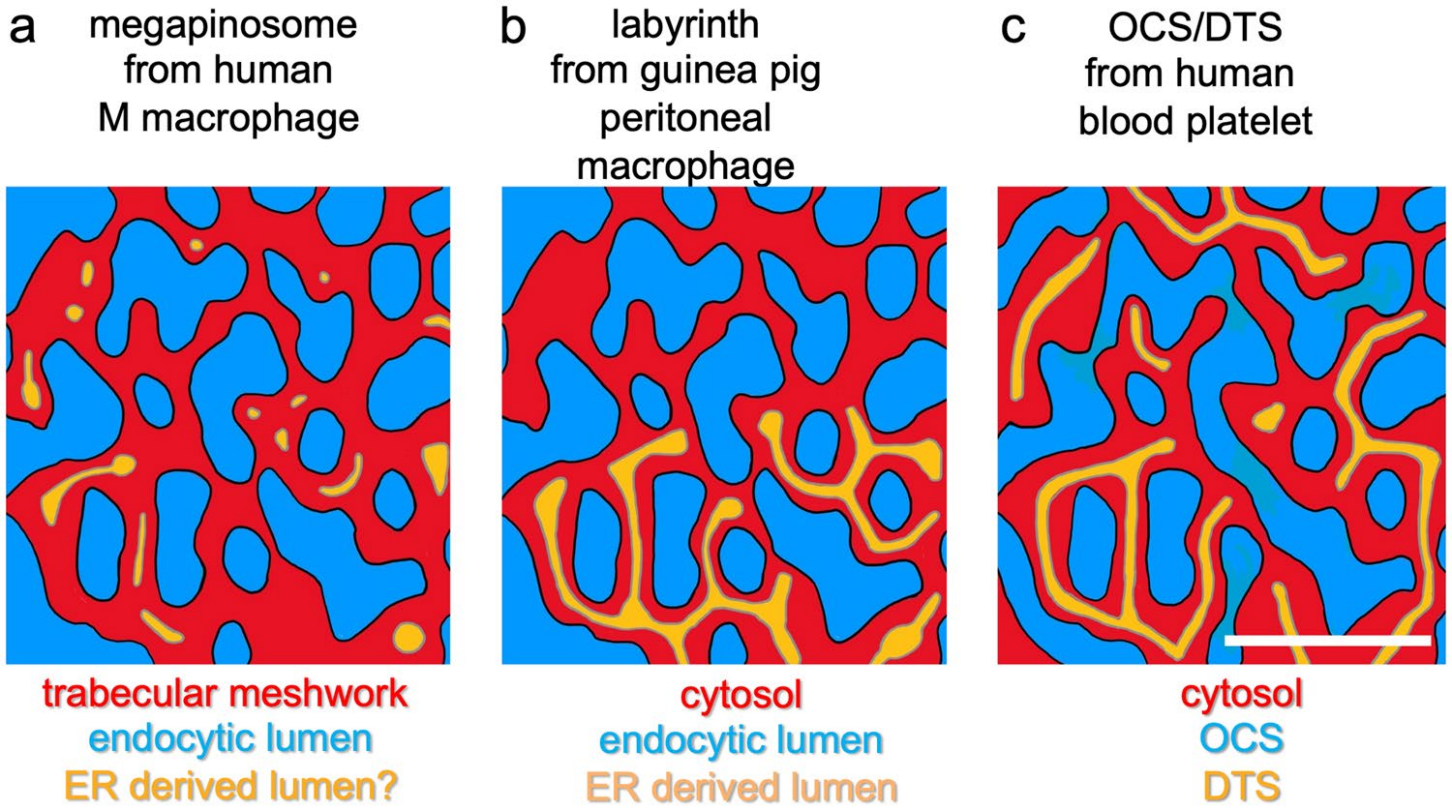
Hypothetical schematic representation of the topological relationship of megapinosomes and homologous structures. Only a portion of the structure is shown, similar to Fig. 1a. The cytosolic parts are red, the endocytic luminal parts are blue, and the most likely endoplasmic reticulum-derived parts are yellow. The membranes to endocytic luminal structure are black, and the membranes to the luminal structure probably homologous to the DTS are grey. **a** An M macrophage’s megapinosome with the cytosolic trabecular meshwork. **b** Homologous structures in peritoneal macrophages from guinea pigs. In contrast to **a**, the endoplasmic reticulum-derived luminal part forms a continuous structure. The same is true for blood platelets in **c**, with the cytosolic parts in red, the OCS in blue, and the DTS in yellow. The scale bar represents 200 nm

Figure 6 shows a schematic representation of a part of the megapinosome and related structures in M macrophages, peritoneal macrophages, and blood platelets. Homologous structures are shown with the same color: first, a megapinosome with the trabecular meshwork (cytosolic area) in red, and the two luminal areas (one endocytic luminal structure in blue, the other luminal structure probably homologous to the DTS in yellow); second a peritoneal macrophage (cytosolic part in red, labyrinth in blue, second luminal structure that is related to the endoplasmic reticulum in yellow); third, the situation in blood platelets with cytosol in red, OCS in blue, and DTS in yellow. The endocytic luminal part and the cytosolic area in peritoneal macrophages (b) and in platelets (c) are morphologically very similar to the megapinosome and the trabecular meshwork. The only difference is the structure shown in yellow. In the megapinosome, these structures are only occasionally found connected. Also, these structures are not found in all bridges and knots in the trabecular meshwork. In the resident guinea pig peritoneal macrophages, the yellow structure forms an interconnected system of tubules. It does not expand through the hole cytosolic part of the labyrinth. In platelets, on the other hand, the yellow structure is represented throughout the cytosolic region as an interconnected tubular network, the DTS.

## Conclusions and outlook

Megapinosome-like structures exist in different hematopoietic cells. It is likely that the structures are homologous. Homologous structures can, but do not necessarily, have the same functions. One known function of the OTS in platelets is to serve as a membrane stock that can be used for the massive surface increases that occur during spreading of the platelets after activation. Thereby the platelets expand and change their shape from discoid to octopus-like, which obviously enhances the surface area. We hypothesized (Bauer et al. 2020) that the membrane stock in megapinosomes could be used by the cell when it changes its shape. When a surface-attached macrophage (that is flattened) detaches and becomes almost spherical, less membrane is required, considering that the volume remains constant. These membranes, which are no longer needed, could be temporarily stored in the megapinosomes.

Another possible function of the megapinosome is filtering. When looking at Fig. 2, the megapinosome looks like a perfect filter between cell lumen and cytosol. The surface between the two topological areas is maximized and would, therefore, be ideal for efficient selected transfer. As mentioned in the Introduction, macrophages indeed absorb large amounts of fluid, clarify it, and release the processed fluid back into the extracellular space (Alberts et al. 2014). Whether megapinosomes are indeed involved in this process needs to be shown.

The biogenesis of megapinosomes, labyrinths, and open canalicular systems remains enigmatic and needs to be approached in future research.

## Supplementary Information

Below is the link to the electronic supplementary material.

Supplementary file 1. Megapinosome complex in 3D, obtained from STEM tomography data. The virtual sections are shown in gray, trabecular meshwork that is part of the cytosol in red, and the luminal part in blue. It is connected with cisterns with a continuous 30-nm gap, as well as with tubules with an average diameter of 165 nm. The trabecular meshwork as well as the luminal part are continuous, meaning that, from each point of the trabecular meshwork (in red), each other point in the trabecular meshwork can be reached without passing through a membrane, and the same is true for the luminal part in blue (MOV 32643 KB)

## Data Availability

All data will be made available upon reasonable request.
